# Implementation of genotype-guided dosing of warfarin with point-of-care genetic testing in three UK clinics: a matched cohort study

**DOI:** 10.1186/s12916-019-1308-7

**Published:** 2019-04-08

**Authors:** Andrea L. Jorgensen, Clare Prince, Gail Fitzgerald, Anita Hanson, Jennifer Downing, Julia Reynolds, J. Eunice Zhang, Ana Alfirevic, Munir Pirmohamed

**Affiliations:** 1 Department of Biostatistics, Institute of Translational Medicine, University of Liverpool, member of Liverpool Health Partners, Liverpool, UK; 2 The Royal Liverpool and Broadgreen University Hospitals NHS Trust and Wolfson Centre for Personalised Medicine, Institute of Translational Medicine, University of Liverpool, Liverpool, UK; 3 Wolfson Centre for Personalised Medicine, Institute of Translational Medicine, University of Liverpool, member of Liverpool Health Partners, Liverpool, UK; 4 Innovation Agency, Academic Health Science Network for the North West Coast, Daresbury, Warrington, UK; 5 MRC Centre for Drug Safety Science, Department of Molecular and Clinical Pharmacology, University of Liverpool and The Royal Liverpool and Broadgreen University Hospitals NHS Trust, members of Liverpool Health Partners, Liverpool, UK; 6 NIHR Collaboration for Leadership in Applied Health Research and Care, North West Coast, UK

**Keywords:** Anticoagulation, Warfarin, Genotype-guided dosing, Pharmacogenetics, Implementation study, Point-of-care

## Abstract

**Background:**

Warfarin is a widely used oral anticoagulant. Determining the correct dose required to maintain the international normalised ratio (INR) within a therapeutic range can be challenging. In a previous trial, we showed that a dosing algorithm incorporating point-of-care genotyping information (‘POCT-GGD’ approach) led to improved anticoagulation control. To determine whether this approach could translate into clinical practice, we undertook an implementation project using a matched cohort design.

**Methods:**

At three clinics (implementation group; *n* = 119), initial doses were calculated using the POCT-GGD approach; at another three matched clinics (control group; *n* = 93), patients were dosed according to the clinic’s routine practice. We also utilised data on 640 patients obtained from routinely collected data at comparable clinics. Primary outcome was percentage time in target INR range. Patients and staff from the implementation group also provided questionnaire feedback on POCT-GGD.

**Results:**

Mean percentage time in INR target range was 55.25% in the control group and 62.74% in the implementation group; therefore, 7.49% (95% CI 3.41–11.57%) higher in the implementation group (*p* = 0.0004). Overall, patients and staff viewed POCT-GGD positively, suggesting minor adjustments to allow smooth implementation into practice.

**Conclusions:**

In the first demonstration of the implementation of genotype-guided dosing, we show that warfarin dosing determined using an algorithm incorporating genetic and clinical factors can be implemented smoothly into clinic, to ensure target INR range is reached sooner and maintained. The findings are like our previous randomised controlled trial, providing an alternative method for improving the risk-benefit of warfarin use in daily practice.

**Electronic supplementary material:**

The online version of this article (10.1186/s12916-019-1308-7) contains supplementary material, which is available to authorized users.

## Background

Warfarin, a vitamin K antagonist, is widely used as an anti-coagulant in the UK and internationally. It is an effective treatment for venous thromboembolism and for prevention of embolic strokes in patients with atrial fibrillation (AF). Warfarin can be challenging to use because of its narrow therapeutic index and inability to predict individual dose requirements, with maintenance doses varying from 0.5 to 20 mg/day [1, 2]. Response to warfarin is monitored using the international normalised ratio (INR), with a target range between 2 and 3 in patients with AF with dose subsequently increased, decreased or maintained depending on INR value. It is important to establish maintenance dose early as it reduces both the risk of complications (bleeding and thrombosis) and the number of clinic visits required for INR monitoring, increasing convenience for both patients and clinics.

Many factors influence warfarin dose requirements, including demographic, clinical and genetic factors [3, 4]. Genetic factors have the greatest influence [5]: two are variants in the *CYP2C9* gene, which is involved in the metabolism of warfarin. Individuals carrying the variants have reduced metabolic capacity with an increased warfarin half-life, therefore requiring a lower dose to achieve a therapeutic INR [6, 7]. The other variant is in the vitamin K epoxide reductase gene, *VKORC1*, an activator of the extrinsic clotting pathway, which warfarin antagonises. Variation in *VKORC1* also influences warfarin dose [6, 7].

Dosing algorithms that predict maintenance warfarin dose requirements of an individual based on a combination of demographic, clinical and genetic factors have been developed [8]. A point-of-care genotype-guided dosing (POCT-GGD) approach based on the algorithm developed by the International Warfarin Pharmacogenetics Consortium (IWPC) [9] was found to be superior to a standard approach to dosing in the European Pharmacogenetics of Anticoagulation Therapy (EU-PACT) trial [10]. The key outcomes from the trial indicated that the POCT-GGD approach (i) improved percentage time within INR target range of 2–3 during the first 3 months of treatment by 7% (95% CI 3–11%), (ii) reduced the proportion of INRs above therapeutic range (odds ratio 0.60, 95% CI 0.41–0.97), and (iii) reduced the time required to achieve target INR (median 21 vs 29 days). A key feature of EU-PACT was the use of a point-of-care genotyping platform, providing genotyping results within 2 h, ensuring there was no delay in initiating warfarin.

In view of this, we undertook an implementation project to determine whether POCT-GGD could translate into routine clinical practice in patients prescribed warfarin for atrial fibrillation (AF) or venous thromboembolism (VTE) in outpatient anticoagulation clinics. In addition to exploring the clinical benefits of adopting a POCT-GGD approach, we were also interested in exploring the practical implications of its adoption from the perspectives of both the staff implementing the approach and of patients.

### Methods

### Project design

Our project is an implementation study using a matched cohort design recruiting patients from six anticoagulation clinics in the North West of England. The study adheres to the StaRI standards for reporting implementation studies [11], and the completed StaRI checklist is attached in Additional file 1. Three of the clinics (implementation group) used POCT-GGD whilst the other three clinics (control group), which were similar to the implementation clinics in terms of patient demographics and clinic organisation, used the standard approach to dosing as per their routine clinical practice.

To improve power for analysing the primary outcome, anonymised data (so-called dashboard data) on INR measurements taken during the first 3 months of treatment for patients attending anticoagulation clinics in the same region were also downloaded from an anticoagulation management software system. The data were from the same period as those from the implementation and control clinics. Dashboard data contributed only to the analysis of outcomes relating to INR measurements, as only data on INR measurements was available for these patients.

### Participants

Each eligible patient, identified as requiring warfarin and attending one of the six clinics for initiation during our recruitment period, was approached during their first clinic visit and given information about the project. Those in the implementation clinics were provided with information about POCT-GGD, were given the option of participating in the project, were given an information sheet and gave their informed consent verbally. Inclusion criteria were that the patient and his/her parents and grandparents were of white ancestry and that they were commencing warfarin due to AF or VTE. Patients with stage 4 (eGFR 15–29 mL/min) or 5 (eGFR < 15 mL/min) chronic kidney disease were excluded.

### Clinic procedures

All patients were recruited from outpatient-based anticoagulation clinics, where they had been referred for commencement onto warfarin. At their first clinic visit (baseline visit), they were provided with verbal information about the study as well as a patient information sheet. Reasons for refusing to participate were recorded on a refusal log.

In the implementation clinics, following agreement to take part, baseline INR was measured and a quality of life EQ-5DL [9] questionnaire completed. In addition, a buccal swab was taken for genotyping and analysed on the point-of-care platform. When genotype results were available, they were input onto a web-based dose calculator programme (‘POCT-GGD calculator’), together with the necessary patient demographics—sex, age, height, weight and amiodarone use. The dose calculator outputs the dose to be taken on days 1, 2 and 3 (loading doses), using the same protocol used in EU-PACT [10]. Patients then returned to clinic on day 4, where their INR was measured and input into the POCT-GGD calculator which output the required dose for days 4 and 5. The POCT-GGD calculator used the same algorithms previously used in EU-PACT. Advice given regarding dosing and INR monitoring from day 6 onwards was decided upon by the treating healthcare professional in accordance with standard clinical care. On day 4, patients were also invited to complete a questionnaire to provide feedback on their experience of POCT-GGD. The questionnaire had six questions and is available in Additional file 2.

In the control clinics, consent was obtained in the same way, and once it had been given, baseline INR was measured and a quality of life EQ-5DL [9] questionnaire completed. However, loading doses, subsequent doses and timings of clinic visits were decided upon entirely according to standard clinical practice.

All patients started their treatment either on the same day as the baseline visit or in the case where the drug had to be prescribed by their GP, within the next few days. Subsequent clinic visits could be either at the hospital clinic or at a community-based clinic.

Data on INR measurements and dose changes during the first 12 weeks of treatment were recorded for all patients, as were details of any hospital admissions or early treatment withdrawals. Patients recruited at all clinics were also contacted by letter or phone 12 weeks after commencing warfarin, during which a quality of life EQ-5DL questionnaire [9] was again completed.

At the end of the study, staff involved in dosing patients at the implementation sites were invited to complete a questionnaire (see Additional file 3) to obtain feedback on POCT-GGD. The questionnaire focussed on three specific aspects of POCT-GGD—administration, training and process—and included five questions on each. There were also free-text boxes where staff could add suggestions on how the process could be improved and provide more general comments on their experience of POCT-GGD.

### ParaDNA point-of-care genotyping platform

Genetic testing was undertaken using the ParaDNA point-of-care genotyping platform developed by LGC (Laboratory of Government Chemist) Limited [12]. It used the same genotyping principles (i.e. HyBeacon probes) as previously used in EU-PACT [10], except that a buccal swab was used to obtain DNA, and the test result was available within 45 min instead of 2 h. No DNA extraction is required for this genotyping platform, with each SNP tested in duplicate reactions, with the ParaDNA data analysis software reporting genotype only if the duplicate results concurred.

For quality assurance, genotyping results from the ParaDNA assays were validated using TaqMan® custom SNP assays. DNA was extracted from each buccal swab using the E.Z.N.A blood mini kit, according to the manufacturer’s protocol (Omega Bio-tek). TaqMan® genotyping of CYP2C9*2 (rs1799853), CYP2C9*3 (rs1057910) and *VKORC1* -1639G → A (rs9923231) was performed on the Applied Biosystems 7900HT Fast Real-Time PCR System. As part of quality control, negative controls containing water instead of DNA and 10% duplicates were included in every run.

### Outcomes assessed and statistical analysis

In order to provide a direct comparison with EU-PACT [10], we evaluated the same outcome measures (primary outcome: percentage time in target INR range during the first 3 months of treatment; secondary outcomes: (i) occurrence of INR ≥ 4 during the first week of treatment, (ii) occurrence of INR < 2 during the first week of treatment, (iii) total number of visits to the clinic during the first 3 months, and (iv) patient adverse events (bleeds, mortality, or other morbidity)). In addition, we also evaluated staff and patient opinions of POCT-GGD. Cost-effectiveness analysis of POCT-GGD implementation was also undertaken and will be reported separately.

A sample size calculation based on EU-PACT findings [10], assuming mean (SD) percentage time in target INR range as 60.3% (21.7%) in the control clinics, required 300 patients in both treatment arms to detect an absolute improvement of 5% in the implementation clinics and achieve 80% power. Assuming a 7% absolute increase, as seen in EU-PACT, this sample size would ensure 98% power.

Due to the decreasing numbers of patients starting warfarin because of increasing use of DOACs, we decided to obtain the anonymised dashboard data, which was available on 640 patients. We assumed a more realistic recruitment target of 100 patients each from both the implementation and control clinics, giving a total of approximately 100 patients dosed according to POCT-GGD and 740 patients dosed according to standard clinical care. Revised power calculations based on these patient numbers showed that assuming a 10% absolute increase in percentage time in range in the POCT-GGD arm we would have 99% power, whilst assuming a 5% absolute increase we would have 58% power. Assuming a 7% absolute increase, as seen in EU-PACT, we would have 86% power.

All statistical analyses were undertaken in R version 3.4.1 [13]. For determining percentage time in target range for each patient, all INR measurements during the first 12 weeks as well as the first INR measurement after the 12-week time point were used, and the method of Rosendaal applied [14]. The number of clinic visits was estimated as the number of INR measurements during the first 12 weeks. For determining whether a patient had INR ≥ 4 or INR < 2 observed during the first week of treatment, all INR measurements during the first week were reviewed, excluding baseline INR.

The primary outcome and secondary outcomes (i)–(iii) were compared between the implementation group and the control group and dashboard data combined (combined control group), as well as between the implementation group and the control group alone. Secondary outcome (iv) could only be compared between the implementation group and control group, since no data on adverse events was available in the dashboard data. Mean percentage time in target range was compared using Student’s *t* test; occurrence of INR ≥ 4 and INR < 2 during the first week and of adverse events during the first 12 weeks of treatment were compared using the Chi-squared test, whilst number of clinic visits during the first 12 weeks of treatment was compared using the Mann-Whitney *U* test. The significance threshold assumed was 0.05.

Primary analysis included patients that remained in the project for at least 2 weeks (14 days). A sensitivity analysis (sensitivity analysis A) was undertaken including only patients who had completed 12 weeks (84 days) of follow-up. For some patients, baseline INR was missing, and for these patients, a baseline INR of one was assumed in the primary analysis. A second sensitivity analysis (sensitivity analysis B) was undertaken, excluding all patients with missing baseline INR.

For the patient and staff questionnaires, a descriptive analysis was undertaken with the proportion of participants choosing each Likert-scale option for each outcome reported. Additional feedback provided by participants in the free-text boxes of the questionnaires was described narratively.

### Results

**Fig. 1:**
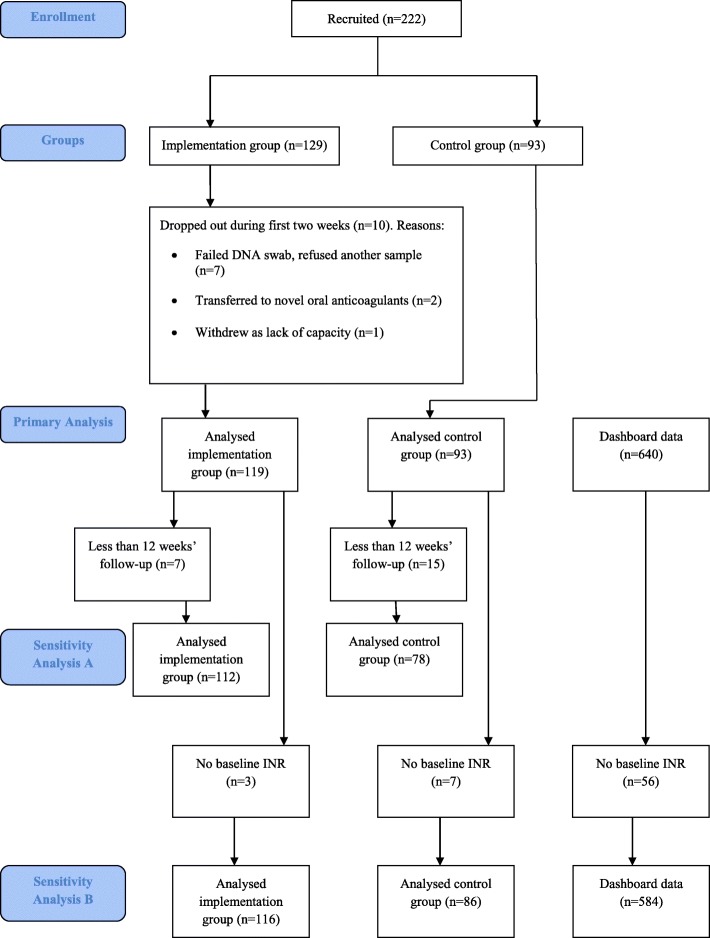
Flowchart to illustrate the number of patients recruited, included and excluded in the analyses

Recruitment commenced in March 2016 and stopped in July 2017, with the final follow-up completed by October 2017. Two hundred and twenty-two patients were recruited, 129 into the implementation group and 93 into the control group. Of the 129 in the implementation group, seven withdrew at baseline due to a failed DNA swab and refusing a second sample. A further patient was withdrawn on day 7 due to lack of capacity whilst two patients transferred onto direct oral anticoagulants during the first 2 weeks. Of the remaining 119, seven had less than 12 weeks’ follow-up and were excluded from sensitivity analysis A. Of the 93 in the control group, 15 patients had less than 12 weeks’ follow-up and were again excluded from sensitivity analysis A. Further details of early withdrawals from both groups are in Fig. 1. Three patients from the implementation group and seven from the control group were excluded from sensitivity analysis B due to missing baseline INRs.

Baseline characteristics of the implementation and control groups are provided in Table 1. No demographic data were available for dashboard data. A slightly higher proportion were men (53.77%), and mean age was 71.21 years. Indication for warfarin treatment was atrial fibrillation for the majority of patients (78.77%), and only a minority (3.30%) were on amiodarone. Self-reported intake of alcohol was less than one unit per week for the majority (58.10%), and just over half were either current (15.64%) or previous smokers (36.02%). The two groups were well balanced with respect to all baseline characteristics apart from indication for treatment—AF was the primary indication for 93.28% of the implementation group but only 59.57% of the control group. Genotype distributions were similar to those previously described [10].

**Table 1:** Baseline characteristics

	Implementation group (*n* = 119)	Control group (*n* = 93)	Total (*n* = 212)
Sex (male)—*n* (%)	63 (52.94)	51 (54.84)	114 (53.77)
Age (years)			
mean	72.14	69.65	71.21
SD	10.67	14.23	12.20
Amiodarone—*n* (%)	3 (2.52)	4 (4.26)	7(3.30)
Alcohol intake (units per day)—*n* (%)			
< 1	62 (52.99)	62(66.67)	122(58.10)
1–5	34(29.06)	21(22.34)	57(27.14)
6–14	10(8.55)	7(7.45)	19(9.05)
15–21	9(7.69)	1(1.06)	10(4.76)
22–49	2(1.71)	2(2.13)	4(1.90)
missing	2		2
Smoking status—*n* (%)			
Current	19(16.10)	14(14.89)	33(15.64)
Previous	39(33.05)	37(39.78)	76(36.02)
Never	60(50.85)	42(44.68)	102(48.34)
Missing	1		1
Indication—*n* (%)			
AF	111 (93.28)	56(59.57)	167(78.77)
DVT	4(3.36)	17(18.09)	21(9.91)
PE	4(3.36)	20(21.51)	24(11.32)
CYP2C9*2—*n* (%)			
*1/*1	91 (76.47)		
*1/*2	27(22.69)		
*2/*2	1(0.84)		
CYP2C9*3—*n* (%)			
*1/*1	105(88.24)		
*1/*3	14(11.76)		
*3/*3	0 (0.00)		
VKORC1—*n* (%)			
G/G	49(41.18)		
G/A	57(47.90)		
A/A	13(10.92)		
Target INR range—*n* (%)			
2–3	115(96.64)	92(98.93)	207(97.64)
2–2.5	0(0.00)	1(1.06)	1(0.47)
2.5–3.5	3(2.52)	0(0.00)	3(1.42)
1.8–3.2	1(0.84)	0(0.00)	1(0.47)

Mean percentage time in range was 62.74% in the implementation group, 54.86% in the control group, 55.31% in the dashboard data and 55.25% in the combined control group. This represents a difference of 7.49 percentage points between the implementation group and combined control group (95% CI 3.41% to 11.57%; *p* = 0.0004) and of 7.89 percentage points (95% CI, 2.25% to 13.522%; *p* = 0.006) between the implementation group and control group alone. Findings of the sensitivity analyses were consistent with those of the primary analysis.

Number of patients with INR ≥ 4 or INR < 2 during the first week of treatment is shown in Table 2. In the implementation group, patients were less likely to exceed INR > 4 (OR 0.21, 95% CI 0.05, 0.89, *p* = 0.03, and OR 0.14, 95% CI 0.03, 0.66, *p* = 0.01; for the combined control group and control group, respectively), whilst an INR < 2 was more common in the implementation group when compared with the combined control group (OR 2.59, 95% CI 1.65, 4.05, *p* < 0.0001), but not with the control group (OR 1.07, 95% CI 0.57, 2.01, *p* = 0.84).

**Table 2:** Outcome data

Outcome	Implementation group	Control group	Dashboard data	Combined control group	Implementation vs combined control		Implementation vs control group	
	*n* = 119	*n* = 93	*n* = 640	*n* = 733	Comparison (95% CI)	*p* value	Comparison (95% CI)	*p* value
Percentage time in therapeutic range—mean % (sd)	62.74 (20.57)	54.86 (20.70)	55.31 (23.10)	55.25 (22.79)	7.49 (3.41 to 11.57)^1^	0.0004 ^6^	7.89 (2.25 to 13.52)^3^	0.006 ^6^
INR ≥ 4 during first week—n (%)	2 (1.68)	10 (10.75)	44 (6.88)	54 (7.37)	0.21 (0.05 to 0.89)^2^	0.03 ^7^	0.14 (0.03 to 0.66)^4^	0.01 ^7^
INR < 2 during first week—*n* (%)	91 (76.47)	70 (75.27)	338 (52.81)	408 (55.66)	2.59 (1.65 to 4.05)^2^	< 0.0001 ^7^	1.07 (0.57 to 2.01)^4^	0.84 ^7^
Number of clinic visits—median (IQR)	10 (8–11)	10 (9–12)	9 (8–13)	10 (8–12)	–	0.49 ^8^	–	0.18 ^8^
Number of adverse events	1^9^	0	N/A	N/A	N/A	N/A	N/A	N/A

^1^Difference in percentage points between implementation and combined control group (implementation minus control)

^2^Odds ratio for implementation group vs combined control group

^3^Difference in percentage points between implementation and control group (implementation minus control)

^4^Odds ratio for implementation group vs control group

^6^*p* value from Student’s t-test

^7^*p* value from Pearson’s chi-square test with Yates’ continuity correction

^8^*p* value from Mann-Whitney U test

^9^One patient stopped warfarin due to bladder bleed

Median number of clinic visits per patient during the first 12 weeks of treatment was not significantly different between groups in either comparison (*p* values of 0.49 and 0.18 for comparison with combined and control groups respectively; see Table 2). One patient in the implementation group stopped warfarin because of a bladder bleed, whilst in the control group, no patients experienced bleeds. There were no other adverse events attributable to either the anticoagulation or genotyping procedure. For all secondary outcomes, the findings of the sensitivity analyses were consistent with those of the primary analysis.

**Fig. 2:**
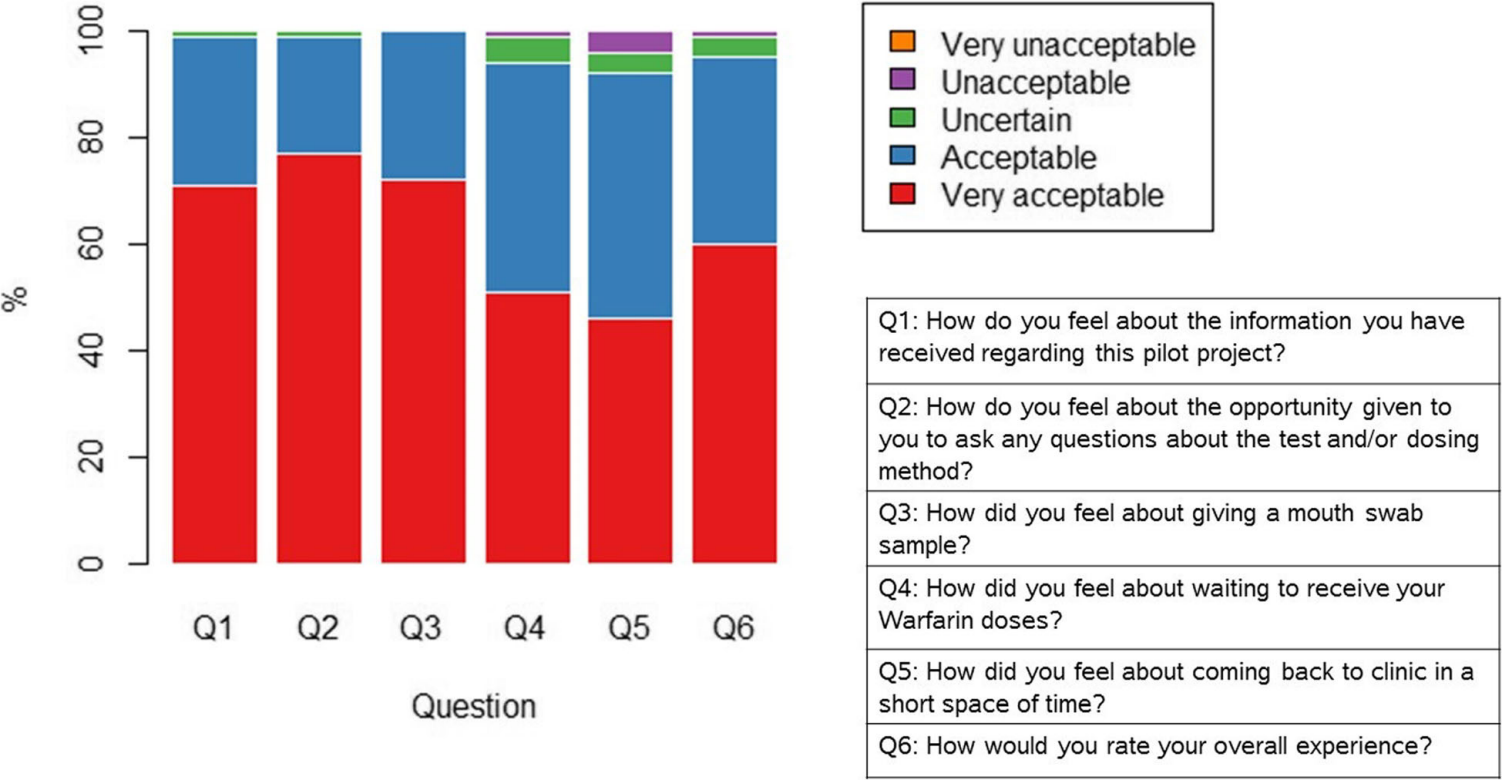
Plot summarising responses to the patient questionnaire (*n* = 114)

Patient questionnaires were completed by 114 patients, and summary results are presented in Table 3 and Fig. 2. The answers demonstrate that the POCT-GGD experience was viewed favourably by the majority, with a rating of ‘Very acceptable’ or ‘Acceptable’ by more than 93% of patients for each question. In the free-text sections, positive comments were made by many patients, including the study and procedures being communicated clearly and staff being helpful and attentive. Only a small number of negative comments were made with two patients commenting that time spent at clinic was too long, another patient stating they found there was a lack of information provided about the procedure, and a further patient commenting that there was lack of communication between the pharmacy and their GP.

**Table 3:** Results of patient questionnaires (*n* = 114)

	Very acceptable (%)	Acceptable (%)	Uncertain (%)	Unacceptable (%)	Very unacceptable (%)
Q1: How do you feel about the information you have received regarding this pilot project?	71	28	1	0	0
Q2: How do you feel about the opportunity given to you to ask any questions about the test and/or dosing method?	77	22	1	0	0
Q3: How did you feel about giving a mouth swab sample?	72	28	0	0	0
Q4: How did you feel about waiting to receive your warfarin doses?	51	43	5	1	0
Q5: How did you feel about coming back to clinic in a short space of time?	46	46	4	4	0
Q6: How would you rate your overall experience?	60	35	4	1	0

**Fig. 3:**
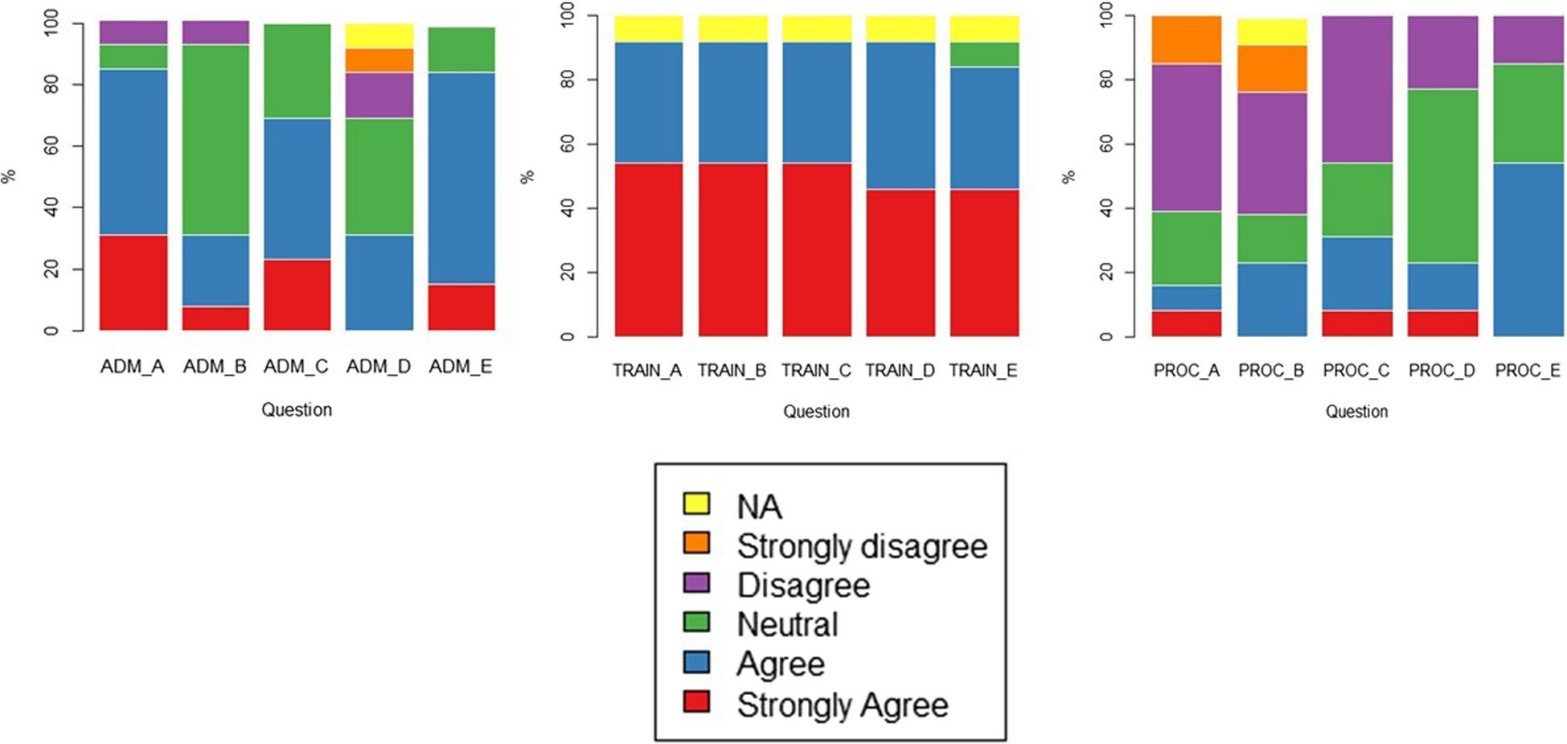
Plot summarising responses to the staff questionnaire (*n* = 13)

Twelve anticoagulation nurses and one healthcare assistant completed the staff questionnaires. Summary results are provided in Table 4 and Fig. 3. Staff responded very positively to the questions about their understanding of sample collection, dose calculation and POCT-GGD initiation procedures and were also positive, overall, about their experience of giving out information about the POCT-GGD procedure and collecting additional information from the patients. However, only 31% of staff agreed that there was enough time to collect additional patient data and patient questionnaires. Further, there was less positivity around the timing of the POCT-GGD process and how it fitted in with the running of the clinic, even though they believed it appeared acceptable to the patients.

**Table 4:** Results of staff questionnaires (*n* = 13)

	Strongly agree	Agree	Neutral	Disagree	Strongly disagree	Not applicable
ADMINISTRATION_A: It was acceptable to give out information to patients in clinic	4 (31%)	7 (54%)	1 (8%)	1 (8%)	0 (0%)	0 (0%)
ADMINISTRATION_B: There was enough time to collect the additional patient data	1 (8%)	3 (23%)	8 (62%)	1 (8%)	0 (0%)	0 (0%)
ADMINISTRATION_C: I felt confident collecting the additional patient data	3 (23%)	6 (46%)	4 (31%)	0 (0%)	0 (0%)	0 (0%)
ADMINISTRATION_D: There was enough time to collect the patient questionnaires	0 (0%)	4 (31%)	5 (38%)	2 (15%)	1 (8%)	1 (8%)
ADMINISTRATION_E: I felt confident collecting the patient questionnaires	2 (15%)	9 (69%)	2 (15%)	0 (0%)	0 (0%)	0 (0%)
TRAINING_A: After training I understand how to collect the mouth swab	7 (54%)	5 (38%)	0 (0%)	0 (0%)	0 (0%)	1 (8%)^1^
TRAINING_B: After training I understand how to transfer cells into the reactor tray using the sample collector	7 (54%)	5 (38%)	0 (0%)	0 (0%)	0 (0%)	1 (8%)^1^
TRAINING_C: After training I understand how to seal and load the reactive tray into the para-DNA machine	7 (54%)	5 (38%)	0 (0%)	0 (0%)	0 (0%)	1 (8%)^1^
TRAINING_D: After training I understand how to use the dose calculator programme	6 (46%)	6 (46%)	0 (0%)	0 (0%)	0 (0%)	1 (8%)^1^
TRAINING_E: After training I felt confident to initiate genotype-guided dosing of warfarin to patients	6 (46%)	5 (38%)	1 (8%)	0 (0%)	0 (0%)	1 (8%)^1^
PROCESS_A: The timing of the genotype-guided dosing system fits in well with the running of the clinic	1 (8%)	1 (8%)	3 (23%)	6 (46%)	2 (15%)	0 (0%)
PROCESS_B: The patients needed to wait or return to clinic after 50 min to receive their warfarin dose and this fitted in well with the running of the clinic	0 (0%)	3 (23%)	2 (15%)	5 (38%)	2 (15%)	1 (8%)
PROCESS_C: Patients returned a few days later to have their warfarin dose adjusted and this fitted in well with the running of the clinic	1 (8%)	3 (23%)	3 (23%)	6 (46%)	0 (0%)	0 (0%)
PROCESS_D: The implementation of genotype-guided dosing for warfarin was worth doing to improve the dosing for patients	1 (8%)	2 (15%)	7 (54%)	3 (23%)	0 (0%)	0 (0%)
PROCESS_E: Overall the genotype-guided dosing of warfarin appeared to be acceptable to our patients	0 (0%)	7 (54%)	4 (31%)	2 (15%)	0 (0%)	0 (0%)

^1^Healthcare assistant not involved in the procedures for which training required

### Validity of genotype results from ParaDNA

For the CYP2C9*2 and *VKORC1* -1639G → A polymorphisms, 100% concordance was observed between the ParaDNA and TaqMan® methods in all samples. Genotype discordance, however, was observed in two samples for CYP2C9*3. The ParaDNA registered two samples as *1*3 whilst the TaqMan® genotype showed *1*1. After investigating the ParaDNA melt curves of these two samples, discrepancy in CYP2C9*3 genotype was observed between the duplicate reactions of each sample, with one showing *1*1, and the other showing *1*3. The ParaDNA software should have registered these two samples as “no call” but erroneously assigned them as *1*3. This was a software glitch which was subsequently corrected in the software's genotype calling algorithm by LGC Limited.

## Discussion

Warfarin can be a challenging drug to use because of its narrow therapeutic index and difficulty in predicting individual dose requirements [1, 2, 15]. For this reason, there has been increasing interest in identifying the genetic and clinical factors responsible for determining variability in anticoagulation with warfarin [5] and the development of dosing algorithms [9]. In the EU-PACT randomised controlled trial, we were able to show that genotype-guided dosing, where dose was predicted using a combination of information on genotype (*CYP2C9* and *VKORC1*), sex, age, height, weight and amiodarone use, was superior to conventional dosing [10]. However, because of strict inclusion and exclusion criteria, the findings of RCTs may not necessarily be generalisable to everyday clinical practice [16] because the patient profiles may be different, and translation to clinical practice is dependent on clinical staff rather than research trained staff. In this paper, we therefore describe an implementation study to determine whether POCT-GGD could be implemented into routine clinical practice in a nurse-led anticoagulant clinic where nurses undertook genotyping (using a point-of-care platform which gave results in 45 min) and dosed according to a web-based algorithm. In order to directly compare with the EU-PACT trial [10], we used the same outcome measures in this implementation study. Our results show that the various parameters we used to assess anticoagulation control improved during routine implementation to the same degree seen in the EU-PACT trial [10]. To our knowledge, this is the first report of implementation of warfarin dosing based on point-of-care genotyping and the use of an algorithm incorporating genetic and clinical factors.

A limitation of our approach is that we have used time in therapeutic INR range rather than the clinical events of bleeding or thrombosis as the outcome measures. Clearly, we did not have the statistical power to assess for differences in the occurrence of clinical events. However, it is important to note that bleeding risk is highly correlated with stability of anticoagulation, with improvement in percentage time in target INR range greater than 10% leading to a 20% improvement in clinical outcomes [17]. Furthermore, analyses of the warfarin arms from the ENGAGE AF-TIMI 48 trial [18] and the Hokusai-venous thromboembolism trial [19] showed that the risk of bleeding was highest in those patients who carried variant alleles for *CYP2C9* and/or *VKORC1* where standard dosing protocols were used rather than genotype-guided dosing. It is also important to note that (a) the 2017 Clinical Pharmacology Implementation Consortium (CPIC) guideline on warfarin prescribing provides detailed genotype-based guidance [20] and (b) the POCT-GGD approach has been shown as cost-effective in the UK and Sweden [21].

A further limitation of our approach was that recruiting patients commencing on warfarin was more difficult than originally predicted, due to the increasing use of DOACs in our clinics. However, we were able to preserve adequate power for testing our primary hypothesis by obtaining the anonymised dashboard data, collected from the same region and time period as the study data.

The POCT-GGD approach was viewed very positively by patients and staff proving it a highly acceptable and relatively easy approach to implement. We acknowledge that the questionnaires developed to obtain patient and staff feedback on POCT-GGD did not undergo validation; nonetheless, we believe that they were able to provide valuable insight into how the approach was perceived by both stakeholder groups. A negative comment from our survey was that almost two thirds of nurses felt that the genotyping approach interfered with the smooth running of the clinic. The genotyping approach requires a discussion with the patients, genotyping which can take 45 min, during which time the patient is waiting, and a further consultation to dose the patients. It can be appreciated that these additional procedures will add to the length of each individual patient consultation and the overall length of the clinic, potentially reducing throughput. This highlights one of the challenges of introducing innovation into clinical practice where disruption of the normal clinical pathway can lead to reduced acceptability of the innovation. A possible solution to this issue may be to separate the genotyping procedure from the anticoagulant clinic consultation, so that the genotype is available at the time of the consultation. Further, provision of genotyping results within a shorter timeframe may be of additional value, and this demonstrates the importance of us continuing to work with industry and ensuring that our research findings inform and support their innovations. As the UK and others develop strategies to increase use of personalised medicine, it is important that lessons learnt from real-world settings within clinics are taken into account as it is likely there will be similar issues with genotype testing in the treatment of other conditions.

Whilst we have been able to replicate the findings of the EU-PACT trial in this implementation study, a criticism of our approach might be that the Clarification of Optimal Anticoagulation through Genetics (COAG) trial did not show any difference between genotyping-guided dosing and the use of a clinical algorithm [22]. However, there were many differences between the EU-PACT [10] and COAG [22] trials, amongst the most significant being that 27% of patients were of African-American origin in the COAG trial, whereas the algorithm utilised largely included polymorphic variants which are prevalent in Caucasian populations [23]. This does however highlight a limitation of our implementation study in that it is relevant to Caucasian patients, and its findings cannot be extrapolated to other ethnic groups. Further work in other ethnic groups will be important to ensure that health inequalities are not exacerbated by the approach we have undertaken. It is also important to note that more recent randomised controlled trials carried out in the USA [24] and Singapore [25] have shown that genotype-guided dosing has clinical utility compared with current clinical practice.

Given the increasing popularity and uptake of the direct acting oral anticoagulants (DOACs), it could be argued that there is no need to undertake studies to improve dosing with warfarin. However, as we have argued previously [26], warfarin is still an important oral anticoagulant, given that there are certain patient sub-groups where DOACs are rarely prescribed and/or contraindicated, including patients on interacting drugs, those with renal impairment or mechanical heart valves and children. Furthermore, whilst DOACs are marketed on the basis of not needing regular monitoring, evidence suggests that a standard dose is not always suitable for all and that many patients may be over- or under-dosed using a universal regimen [27]. Finally, although the use of DOACs has been shown to be cost-effective, the actual cost outlay for DOACs is huge, with expenditure on anticoagulants rising by around £400 million in 2016/17 and predicted to rise to £1 billion per year by 2020 [28].

## Conclusions

In conclusion, therefore, there is a continuing clinical need for warfarin, and it is imperative that those patients prescribed warfarin are prescribed the correct dose from the outset, to ensure target INR is achieved rapidly and is maintained. We have demonstrated in this implementation study that the POCT-GGD approach not only achieves this aim, but does so in a way that is viewed positively, overall, by patients and staff. More widespread implementation of genotype-guided dosing of warfarin should be considered where it remains the best oral anticoagulation option.

## Additional files

Additional file 1: StaRI checklist. (DOCX 62 kb)

Additional file 2: Patient questionnaire. (DOCX 26 kb)

Additional file 3: Staff questionnaire. (DOCX 131 kb)

Additional file 4: Study data. (XLSX 66 kb)

## Abbreviations

AF: Atrial fibrillation
COAG trial: Clarification of Optimal Anticoagulation through Genetics trial
CPIC: Clinical Pharmacology Implementation Consortium
DOACS: Direct acting oral anticoagulants
EU-PACT: European Pharmacogenetics of Anticoagulation Therapy trial
INR: International normalised ratio
IWPC: International Warfarin Pharmacogenetics Consortium
POCT-GGD: Point-of-care testing genotype-guided dosing
VTE: Venous thromboembolism

## References

[1] Burns M (1999). Management of narrow therapeutic index drugs. J Thromb Thrombolysis.

[2] Gong IY, Schwarz UI, Crown N, Dresser GK (2011). Clinical and genetic determinants of warfarin pharmacokinetics and pharmacodynamics during treatment initiation. PLoS One.

[3] Johnson JA, Gong L, Whirl-Carrillo M, Gage BF (2011). Clinical Pharmacogenetics Implementation Consortium guidelines for CYP2C9 and VKORC1 genotypes and warfarin dosing. Clin Pharmacol Ther.

[4] Wadelius M, Pirmohamed M (2007). Pharmacogenetics of warfarin: current status and future challenges. Pharmacogenomics J.

[5] Bourgeois S, Jorgensen A, Zhang EJ, Hanson A (2016). A multi-factorial analysis of response to warfarin in a UK prospective cohort. Genome Med.

[6] Jorgensen AL, FitzGerald RJ, Oyee J, Pirmohamed M (2012). Influence of CYP2C9 and VKORC1 on patient response to warfarin: a systematic review and meta-analysis. PLoS One.

[7] Yang J, Chen Y, Li X, Wei X (2013). Influence of CYP2C9 and VKORC1 genotypes on the risk of hemorrhagic complications in warfarin-treated patients: a systematic review and meta-analysis. Int J Cardiol.

[8] Francis B, Lane S, Pirmohamed M, Jorgensen A (2014). A review of a priori regression models for warfarin maintenance dose prediction. PLoS One.

[9] Consortium TIWP (2009). Estimation of the Warfarin dose with clinical and Pharmacogenetic data. N Engl J Med.

[10] Pirmohamed M, Burnside G, Eriksson N, Jorgensen AL (2013). A randomized trial of genotype-guided dosing of warfarin. N Engl J Med.

[11] Pinnock H, Barwick M, Carpenter CR, Eldridge S (2017). Standards for Reporting Implementation Studies (StaRI) statement. BMJ.

[12] www.lgcgroup.com. Accessed 14 Mar 2019.

[13] R Development Core Team (2010). R: A language and environment for statistical computing.

[14] Rosendaal FR, Cannegieter SC, van der Meer FJ, Briet E (1993). A method to determine the optimal intensity of oral anticoagulant therapy. Thromb Haemost.

[15] Lee MTM, Klein TE (2013). Pharmacogenetics of Warfarin: challenges and opportunities. J Hum Genet.

[16] Rothwell PM (2005). External validity of randomised controlled trials: “to whom do the results of this trial apply?”. Lancet.

[17] Van Spall HG, Wallentin L, Yusuf S, Eikelboom JW (2012). Variation in warfarin dose adjustment practice is responsible for differences in the quality of anticoagulation control between centers and countries: an analysis of patients receiving warfarin in the randomized evaluation of long-term anticoagulation therapy (RE-LY) trial. Circulation.

[18] Mega JL, Walker JR, Ruff CT, Vandell AG (2015). Genetics and the clinical response to warfarin and edoxaban: findings from the randomised, double-blind ENGAGE AF-TIMI 48 trial. Lancet.

[19] Vandell AG, Walker J, Brown KS, Zhang G (2017). Genetics and clinical response to warfarin and edoxaban in patients with venous thromboembolism. Heart.

[20] Johnson JA, Caudle KE, Gong L, Whirl-Carrillo M (2017). Clinical Pharmacogenetics Implementation Consortium (CPIC) guideline for pharmacogenetics-guided warfarin dosing: 2017 update. Clin Pharmacol Ther.

[21] Verhoef TI, Redekop WK, Langenskiold S, Kamali F (2016). Cost-effectiveness of pharmacogenetic-guided dosing of warfarin in the United Kingdom and Sweden. Pharmacogenomics J.

[22] Kimmel SE, French B, Kasner SE, Johnson JA (2013). A pharmacogenetic versus a clinical algorithm for warfarin dosing. N Engl J Med.

[23] Pirmohamed M, Kamali F, Daly AK, Wadelius M (2015). Oral anticoagulation: a critique of recent advances and controversies. Trends Pharmacol Sci.

[24] Gage BFBA, Lin H, Woller SC, Stevens SM, Al-Hammadi N, Li J, Rodríguez T, Miller JP, McMillin GA, Pendleton RC, Jaffer AK, King CR, Whipple BD, Porche-Sorbet R, Napoli L, Merritt K, Thompson AM, Hyun G, Anderson JL, Hollomon W, Barrack RL, Nunley RM, Moskowitz G, Dávila-Román V, Eby CS (2017). Effect of genotype-guided warfarin dosing on clinical events and anticoagulation control among patients undergoing hip or knee arthroplasty: the GIFT randomized clinical trial. JAMA.

[25] Syn NL, Wong AL, Lee SC, Teoh HL (2018). Genotype-guided versus traditional clinical dosing of warfarin in patients of Asian ancestry: a randomized controlled trial. BMC Med.

[26] Pirmohamed M (2018). Warfarin: the end or the end of one size fits all. J Personalized Med.

[27] Gulilat M, Tang A, Gryn SE, Leong-Sit P (2017). Interpatient variation in rivaroxaban and apixaban plasma concentrations in routine care. Can J Cardiol.

[28] Burn J, Pirmohamed M (2018). Direct oral anticoagulants versus warfarin: is new always better than the old? (vol 5, year 2018). Open Heart.

