# Supplementary material for: Implementation of genotype-guided dosing of warfarin with point-of-care genetic testing in three UK clinics: a matched cohort study
Source: BMC Med. 2019 Apr 8;17:76. doi: 10.1186/s12916-019-1308-7 (PMC6454722; doi:10.1186/s12916-019-1308-7)
Supplement: Supplementary file 2 — Patient questionnaire. (DOCX 26 kb) [file 12916_2019_1308_MOESM2_ESM.docx]

**Additional file 2: Patient Questionnaire**

| **FOR OFFICE USE ONLY:** | |
| --- | --- |
|  |  |
| Site Code: |  |
|  |  |
| Site Number: |  |

**Genotype Guided Dosing Pilot Project**

**Patient Questionnaire**

We are continually trying to improve our services and any information that you can give us by completing this questionnaire will help us with this. Any information you give will be treated in the strictest confidence.

| **You have attended the hospital to be started on Warfarin Medication; so that we give the best dose of medication to each person we have introduced a new way of testing people.**  **1. How do you feel about the information you have received regarding this pilot project?**  Very Acceptable  Acceptable  Uncertain  Unacceptable  Very Unacceptable  **2. How do you feel about the opportunity given to you to ask any questions about the test and/or dosing method?**  Very Acceptable  Acceptable  Uncertain  Unacceptable  Very Unacceptable  **3. How did you feel about giving a mouth swab sample?**    Very Acceptable  Acceptable  Uncertain  Unacceptable  Very Unacceptable  **4. How did you feel about waiting to receive your Warfarin doses?**  Very Acceptable  Acceptable  Uncertain  Unacceptable  Very Unacceptable  **5. How did you feel about coming back to clinic in a short space of time?**  Very Acceptable  Acceptable  Uncertain  Unacceptable  Very Unacceptable  **6. How would you rate your overall experience?**  Excellent  Very Good  Good  Fair  Poor  **7. Was there anything particularly good about your hospital care?**  **8. Is there anything that could have been improved?**  **9. Anything else you want to tell us about your experience please write here.**  **10. Would you like to take part in a telephone interview to tell us more about your experience?**  Yes  No  **11. Would you like to take part in a group discussion with other patients about your experience?**  Yes  No  *If you said yes, please contact* ***Jenny Downing or Gail Fitzgerald*** *on telephone number*  ***0151 794 5539*** *so we can contact you to arrange an interview or group discussion.* |
| --- |

The information collected about you will be anonymised and transferred to The University of Liverpool. It will be stored securely and only kept for the duration of the project.

**THANK YOU FOR TAKING TIME TO COMPLETE THIS QUESTIONNAIRE**
