# Supplementary figures and images for: Implementation of genotype-guided dosing of warfarin with point-of-care genetic testing in three UK clinics: a matched cohort study
Source: BMC Med. 2019 Apr 8;17:76. doi: 10.1186/s12916-019-1308-7 (PMC6454722; doi:10.1186/s12916-019-1308-7)

**Additional file 3: Staff Questionnaire**


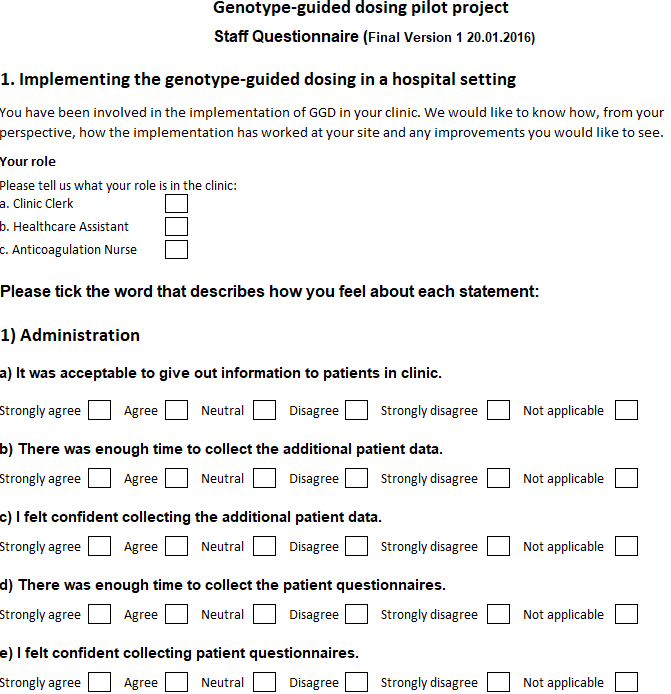


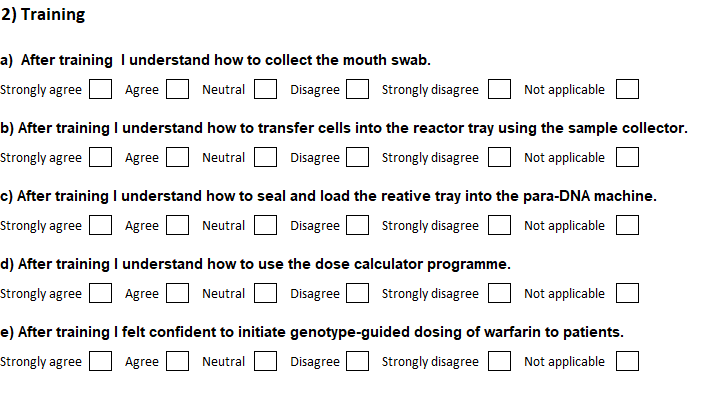


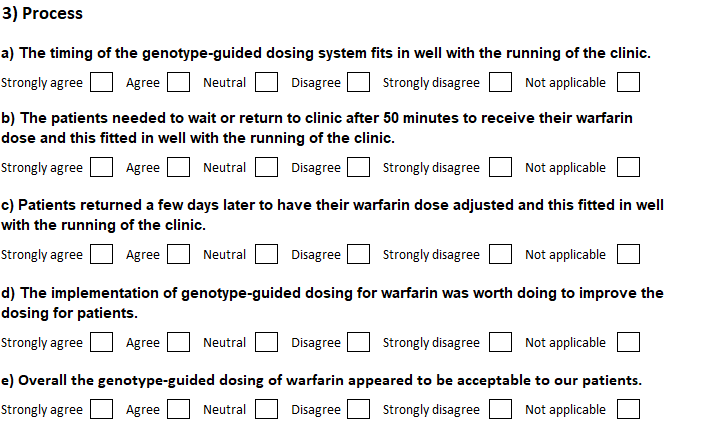


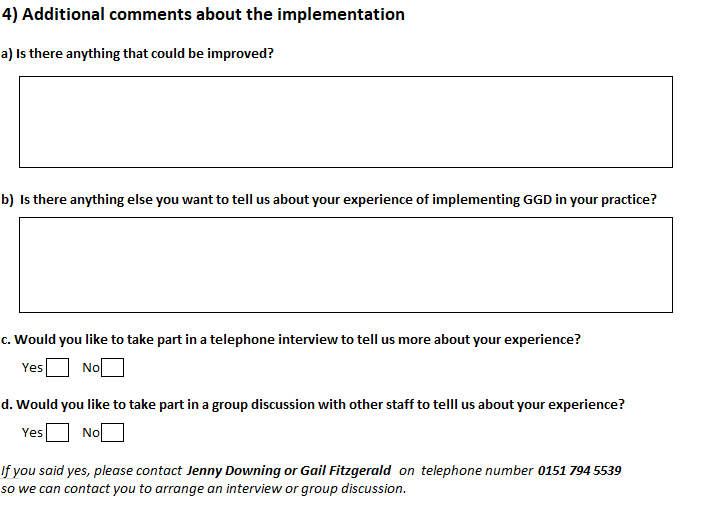

Supplement: Supplementary file 3 — Staff questionnaire. (DOCX 131 kb) [file 12916_2019_1308_MOESM3_ESM.docx]
